# α-Synuclein Interacts with Lipoproteins in Plasma

**DOI:** 10.1007/s12031-017-0967-0

**Published:** 2017-09-08

**Authors:** Fatemeh Nouri Emamzadeh, David Allsop

**Affiliations:** Division of Biomedical and Life Sciences, Faculty of Health and Medicine, University of Lancaster, Lancaster, LA1 4AY UK

**Keywords:** Parkinson’s disease (PD), Blood-brain barrier (BBB), α-Synuclein (α-syn), Lipoproteins

## Abstract

Parkinson’s disease (PD) is an age-related neurodegenerative disorder characterized by dopaminergic neural cell death in the substantia nigra of the brain and α-synuclein (α-syn) accumulation in Lewy bodies. α-Syn can be detected in blood and is a potential biomarker for PD. It has been shown recently that α-syn can pass through the blood-brain barrier (BBB), but the mechanism is not yet understood. We hypothesized that α-syn could interact with lipoproteins, and in association with these particles, could pass through the BBB. Here, we show that apoE, apoJ, and apoA1, but not apoB, were co-immunocaptured along with α-syn from human blood plasma, suggesting that α-syn is associated with high-density lipoproteins (HDL). This association was also supported by experiments involving western blotting of plasma fractions separated by gel filtration, which revealed that α-syn was found in fractions identified as HDL. Interestingly, we could also detect α-syn and ApoJ in the intermediate fraction between HDL and LDL, referred to as lipoprotein (a) (Lp(a)), which has an important role in cholesterol metabolism. Overall, the results provide best support for the hypothesis that α-syn interacts with HDL, and this has potential implications for transport of α-syn from the brain to peripheral blood, across the BBB.

## Introduction

Parkinson’s disease (PD) is a progressive brain disorder, the main neuropathological feature of which is the degeneration of dopaminergic neurons in the substantia nigra (SN) pars compacta of the brain, resulting in tremor, muscle stiffness, and other motor and non-motor defects (Jankovic 2008). In this disease, a protein called α-synuclein (α-syn) accumulates in an aggregated and fibrillar form within the Lewy bodies (LBs), which are found in surviving neurons of the SN and other brain regions (Spillantini et al. 1997). LBs are also present, but in more widespread areas of the brain, in dementia with Lewy bodies (DLB) (Spillantini et al. 1998).

It is now clear that α-syn can be secreted from cells into the extracellular space and is found in human body fluids, including the cerebrospinal fluid (CSF) and blood plasma (El-Agnaf et al. 2003; Mollenhauer et al. 2011). This has led to considerable interest in α-syn as a potential biomarker for PD and/or other α-synucleinopathies (El-Agnaf et al. 2006; Hong et al. 2010; Foulds et al. 2011, 2013). Once initiated, the aggregation of α-syn appears to spread throughout adjacent parts of the brain by propagation in a “prionoid” fashion (Alvarez-Erviti et al. 2011; Olanow and Brundin 2013; Aulić et al. 2014; Narkiewicz et al. 2014). These α-syn aggregates may be harmful to dopaminergic neurons in the SN, especially when α-syn is present in the form of toxic oligomers (Kalia et al. 2013).

It is well known that α-syn interacts with lipids, resulting in its transition from a natively unfolded protein into a more helical structure (Auluck et al. 2010). Therefore, it is conceivable that α-syn is associated with lipoprotein particles (such as VLDL, LDL, HDL, VHDL) in blood and in other tissues. If so, this would have important implications for the study of α-syn as a potential biomarker, as well as for understanding its normal function and its role in disease. Apolipoproteins have 11 amino acid repeats that mediate lipid interactions in a similar way to the N-terminal α-helices of α-syn. Like α-syn, apolipoproteins, with their amphipathic helices, can insert into lipid membranes and influence their curvature (Varkey et al. 2010).

A potential association between α-syn and lipoproteins is also emerging from other observations. It is thought that α-syn plays an important role in synaptic vesicle recycling at the dopaminergic synapse (Busch et al. 2014; Vargas et al. 2014), and the involvement of lipoproteins could provide a source of the negatively charged lipids required for expansion of synaptic membranes during formation and release of synaptic vesicles (Eichmann et al. 2016). Furthermore, α-syn-lipoprotein interactions could also be involved in the transport of α-syn across the blood-brain barrier (BBB). An imbalance in the flux of the β-amyloid (Aβ) protein between the brain and blood, resulting in reduced brain amyloid clearance, has already been implicated as a possible causative event in Alzheimer’s disease (Tarasoff-Conway et al. 2015), and the same could be true for α-syn in PD and other α-synucleinopathies. Indeed, it has been determined already that radiolabeled α-syn can enter the CNS from the blood, and vice versa, implying its passage across the BBB (Sui et al. 2014). It is not yet clear whether this BBB transport depends on the intermediation of specific receptors or if α-syn can simply diffuse across the BBB based on its amphipathic properties (Sui et al. 2014; Steiner et al. 2011). Also, it has been shown that deletion of *SNCA* in knockout mice causes an increased level of brain cholesterol, suggesting that α-syn is involved in the transport of cholesterol out of the brain (Barceló-Coblijn et al. 2007).

Here, we hypothesize that α-syn is carried in the blood and/or CSF in association with lipoproteins, and that this could aid its passage across the BBB. To investigate whether α-syn exists in association with lipoproteins in blood plasma, we used different methods including immunoprecipitation, SDS-PAGE/immunoblotting, plasma fractionation by gel filtration, and immunoassay. The experimental data support the idea that α-syn is found in association with HDL in human blood.

## Material and Methods

### Human Plasma Samples for α-Synuclein/Apolipoprotein Study

About 5 ml of blood was collected from two healthy males, 24 and 27 years old, and one female, 25 years old, in tubes containing EDTA to prevent clotting. The separation of plasma was achieved within 2 h of blood collection by centrifugation at 3000*g* and 15 °C for 10 min to avoid red blood cell (RBC) rupture and hemolysis as a consequence of exposure to excessive heat or cold. Then, 1.5 ml of plasma was transferred to plastic tubes containing a protease inhibitor cocktail consisting of 104 mM AEBSF, 80 μM aprotinin, 4 mM bestatin, 1.4 mM E64, 2 mM leupeptin, and 1.5 mM pepstatin A (Sigma P8340), and stored at − 80 °C. Moreover, to reduce hemolysis and destruction of RBC membrane, vigorous mixing or shaking was avoided. The samples were thawed at room temperature directly before analysis without repeated freeze/thaw cycles.

### Crosslinking of Anti-α-syn Antibody to Dynabeads

The beads (Pierce protein A/G magnetic beads with binding capacity 55 to 85 μg rabbit IgG per mg magnetic particles) were vortexed for 1–2 min to resuspend them in solution. Then, 100 μl of beads was transferred to a 1.5-ml plastic tube. The tube was placed in a Dynal magnetic particle concentrator (MPC) for 1 min, and the fluid was removed. The beads were washed three times with 0.5 ml of 0.1 M phosphate buffer, pH 8.2. Four hundred microliters of 80 μg antibody was added to the tube containing the beads, and incubated for 15–30 min with mixing by rotation at RT. These antibodies are Santa Cruz anti-α-syn C211, sc-58480; Millipore anti-apolipoprotein A1, AB740; Millipore anti-apolipoprotein B, AB742; Millipore anti-apolipoprotein B, AB947 and abcam anti-apolipoprotein J, ab7621. The beads were washed three times with 0.5 ml of 0.1 M phosphate buffer, pH 8.2 and washed again two times with 1 ml of 0.2 M triethanolamine, pH 8.2. Then, fresh 20 mM DMP (dimethyl pimelinediimidate dihydrochloride) solution was prepared and 1 ml of it was added to the beads and incubated for 30 min on the mixing rotator, at RT. The reaction was stopped by removing the DMP buffer from the beads and by adding 1 ml of 50 mM Tris-HCl, pH 7.4. The tube was mixed and incubated for 30 min on the rotator at RT. The beads were washed three times with 0.5 ml PBS and washed again twice with 0.5 ml citrate buffer pH 3.1 and placed on the mixing rotator for 2 min each time. Finally, the beads were washed three times with PBS and stored in 0.5 ml PBS. For long-term preservation, the beads were stored in PBS containing 0.05% NaN_3_ at 4 °C.

### Immunoprecipitation

Fifty microliters of antibody-conjugated beads was transferred to a tube at RT. The tube was placed on the MPC for 1–2 min, and the supernatant discarded. The beads were washed three times with 0.1 M phosphate buffer, pH 8.2. One milliliter of plasma was added to the beads. The tube was incubated for 1 h on the mixing rotator at RT and then was placed on the MPC for 2 min and the supernatant discarded. Finally, the beads were washed up to seven times with PBS.

### Elution Procedure

For mild elution, 40 μl of 0.1 M citrate buffer, pH 3, was added to the beads and incubated for 2 min on the mixing rotator at RT. The tube was placed on the magnet, and the supernatant, containing antigen, was transferred to a clean tube. The sample was then adjusted to physiological pH by adding 1 M Tris, pH 7.5.

For strong elution, the beads were boiled for 10 min in SDS gel loading buffer. The tube was placed on the magnet, and the supernatant, containing antigen, was transferred to a clean tube.

### Immunoblotting

Fifteen percent acrylamide/bis-acrylamide gel was made using Sigma, A3699. A relevant molecular weight marker and the samples were loaded in the wells. The gel was run at 180 V for 1 h in running buffer containing 25 mM Tris, 192 mM glycine, and 0.1% SDS, pH ~ 8.5. The separated proteins were transferred to a nitrocellulose membrane by wet transfer at 25 V for 1 h in transfer buffer containing 25 mM Tris, 192 mM glycine, and 20% methanol. Afterwards, the membrane was incubated in 25 ml of 5% skimmed milk as blocking buffer for 1–2 h at room temperature, following by an optional wash of the membrane with PBS-T (10 mM sodium phosphate, 0.15 M NaCl, 0.05% Tween-20, pH 7.5). The membrane was then incubated with the primary antibody (at the appropriate dilution) in 10 ml of 5% skimmed milk in PBS-T with gentle agitation overnight at 4 °C. To remove unbound antibodies, the membrane was washed three times for 30 min each with PBS-T. The membrane was incubated with the secondary antibody, horseradish peroxidase (HRP) conjugated to goat anti-rabbit (DAKO, K1497), rabbit anti-goat (DAKO, P0449), or goat anti-mouse (DAKO, K4000) antibody, as appropriate, diluted in PBS-T. The membrane was incubated with the secondary antibody with gentle agitation for 45 min at RT followed by three washes of 30 min each with PBS-T. Finally, 1 ml of luminol/enhancer buffer and stable peroxide buffer (SuperSignal West Pico Chemiluminescent Substrate, Pierce 34079) was applied onto the membrane for 3 min. Then, the membrane was drained of excess developing solution (without drying completely), wrapped in plastic film, and exposed to X-ray film.

### Gel Filtration Analysis of Plasma Lipids

Blood was collected into the EDTA tube to stop clotting after a 4–5-h fast from a healthy volunteer (35 years old, male). Plasma was separated by centrifugation for 10 min at 1000*g* and 0.2 ml of plasma was loaded immediately onto two Superose-6 10/300GL columns connected in series to an ÄKTA purifier system (GE Healthcare, MD, USA). Fractions of 0.5 ml were collected using an elution buffer (1 mM EDTA, 154 mM NaCl, and 0.02% NaN3, pH 8.2) at a flow rate of 0.5 ml/min. The lipid contents (triglyceride, cholesterol) in individual fractions were determined with enzymatic assay kits (Chang et al. 1999). The size-fractionated samples of human plasma were prepared, with appropriate ethical approval from the donor concerned, by one of the collaborators in Japan (Dr. Takashi Kasai, Department of Neurology, Kyoto Prefectural University of Medicine).

### Immunoassay for Measuring Total α-Syn

An enzyme-linked immunosorbent assay (ELISA) plate was coated with 150 μl/well of anti-α-syn C211 antibody (Santa Cruz, sc-12767), diluted 1 μg/ml in 200 mM NaHCO3, pH 9.6, and incubated at 4 °C overnight. The wells were then washed four times with PBS containing 0.05% Tween-20 (PBS-T), and incubated for 2 h at 37 °C with 200 μl/well of freshly prepared blocking buffer 2.5% fish gelatin, Sigma G7765, in PBS-T. The plate was washed again with PBS-T. One hundred fifty microliters of plasma fraction samples was added to each well, and the assays were performed in triplicate. Following this, the plate was incubated at 37 °C for 2 h. After a repeat wash with PBS-T, 100 μl/well of the detection antibody, anti-α/β/γ-synuclein FL140 antibody (Santa Cruz, sc-10717), dilution 1:1000 in blocking buffer, was added, and the plate incubated at 37 °C for 2 h. After another wash with PBS-T, the plate was incubated with 100 μl/well of the secondary antibody (goat anti-rabbit HRP, dilution 1:10,000) in blocking buffer at 37 °C for 2 h. The plate was then washed again with PBS-T before adding 100 μl/well of Sure Blue TMB Microwell Peroxidase Substrate (KPL, USA) and leaving the color to develop for 30 min at RT. Finally, 100 μl/well of stop solution was added and absorbance at 450 nm determined.

### Production of Recombinant α-Synuclein Protein

Recombinant α-syn (R-α-syn) protein was produced by using a pET11a expression vector introduced into *Escherichia coli* (Emamzadeh et al. 2016).

## Results

**Fig. 1:**
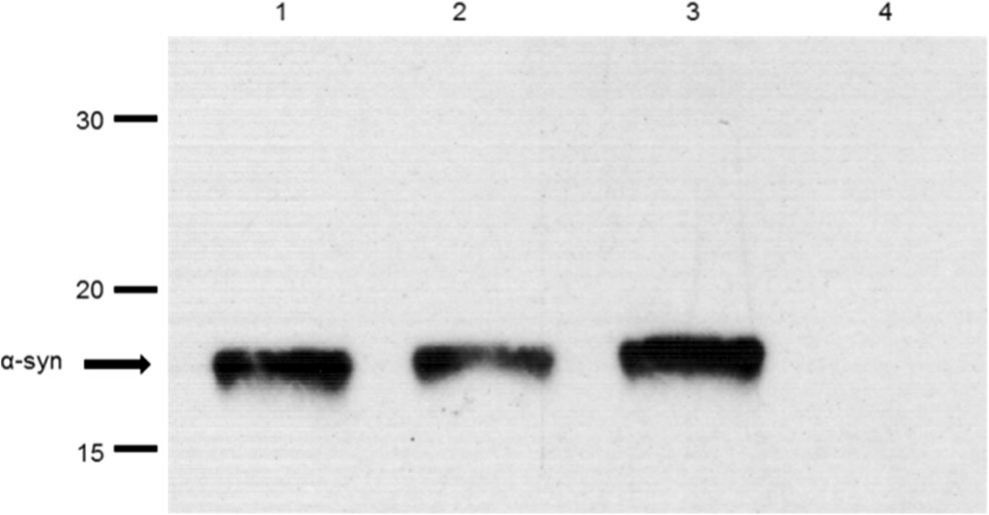
Immunocapture of α-syn by C211-dynabeads. Plasma, R-α-syn (positive control), or PBS (negative control) were incubated with magnetic dynabeads coupled to C211 anti-α-syn antibody (“beads + Ab”). Whole plasma was also incubated with empty “beads” (without any antibody). The immunoprecipitants were run on a 15% SDS-PAGE gel and immunoblotted with FL140 antibody (1:1500). Lane 1 = 50 ng of R-α-syn; Lane 2 = eluant from beads exposed to R-α-syn; Lane 3 = eluant from beads exposed to plasma; Lane 4 = eluant from unconjugated beads exposed to plasma

**Fig. 2:**
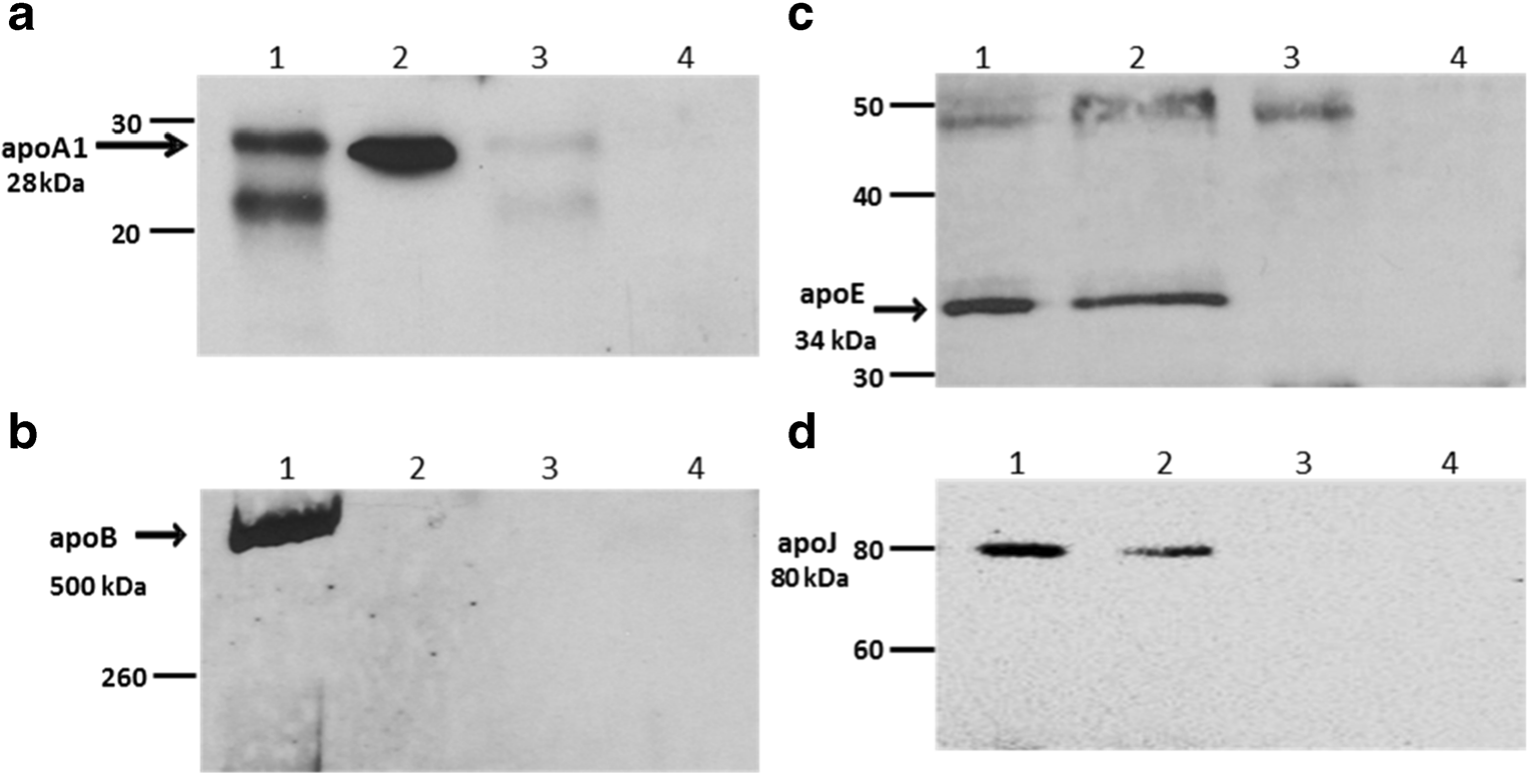
Detection by SDS-PAGE/immunoblotting of apolipoproteins in material captured from plasma by dynabeads cross-linked with anti-α-syn antibody C211. **a** Mild elution by citrate followed by immunoblotting with anti-apoA1. **b** Elution by SDS followed by immunoblotting with anti-apoB. **c** Elution by SDS followed by immunoblotting with anti-apoE. **d** Elution by SDS followed by immunoblotting with anti-apoJ. In each case: Lane 1 = apolipoprotein standard; Lane 2 = eluant from beads exposed to plasma; Lane 3 = eluant from unconjugated beads exposed to plasma; Lane 4 = eluant from conjugated beads exposed to PBS. Numbers on LHS indicate positions of MW standards (in kDa). Bands corresponding to apoA1, apoB, apoE, and apoJ are also indicated

To investigate whether α-syn interacts with lipoproteins in human blood plasma, a sample of plasma from a healthy individual was incubated with protein A/G dynabeads cross-linked with anti-α-syn C211 antibody, and, after extensive washing, the resulting immunocaptured material was eluted from the beads, fractionated on 15% SDS-PAGE gels, and immunoblotted to detect α-syn (Fig. 1) as well as apolipoproteins including apoA1 (Fig. 2a), apoB (Fig. 2b), apoE (Fig. 2c), and apoJ (Fig. 2d). In each case, samples of whole plasma, diluted 1/300 in PBS, were also included on the gels as a positive control for detection of each of these apolipoproteins. Plasma was also incubated with unconjugated beads as a control for non-specific binding of plasma proteins to the beads in the absence of C211 capture antibody. Antibody-coupled beads were also incubated with PBS to control for elution of non-plasma components (e.g., C211 heavy and light chains) from the beads. The results, presented in Fig. 2, show that apoA1 (28 kDa), apoE (34 kDa), and apoJ (80 kDa) were co-eluted with α-syn, but apoB (500 kDa) was not detected in the eluant from the beads. ApoB was, however, easily detected in whole plasma (Fig. 2b). There was no evidence for non-specific binding of any of these apolipoproteins to unconjugated beads. Therefore, the results show that apoE, apoA1, and apoJ, but not apoB, were captured from plasma along with α-syn.

**Fig. 3:**
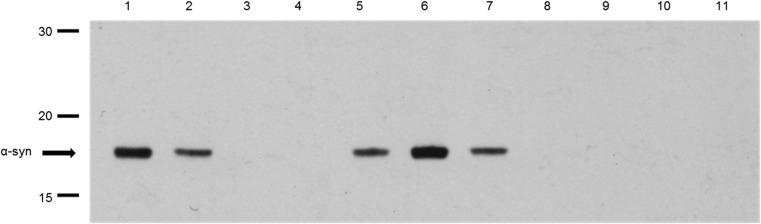
Detection of α-syn by anti-α-syn antibody C211in material captured from plasma by dynabeads cross-linked with anti-apolipoprotein antibodies. Lane 1 = 50 ng of R-α-syn as positive control; Lane 2 = whole plasma as a positive control; Lane 3 = eluant from unconjugated beads exposed to plasma as a negative control; Lane 4 = eluant from conjugated beads with anti-apoB antibody exposed to plasma. Lane 5 = eluant from conjugated beads with anti-apoA1 antibody exposed to plasma. Lane 6 = eluant from conjugated beads with anti-apoJ antibody exposed to plasma. Lane 7 = eluant from conjugated beads with anti-apoE antibody exposed to plasma. Lane 8 = eluant from conjugated beads with anti-apoB antibody exposed to PBS. Lane 9 = eluant from conjugated beads with anti-apoA1 antibody exposed to PBS. Lane 10 = eluant from conjugated beads with anti-apoJ antibody exposed to PBS. Lane 11 = eluant from conjugated beads with anti-apoE antibody. Numbers on LHS indicate positions of MW standards (in kDa). Bands corresponding to α-syn are also indicated

To confirm these results, plasma samples were incubated with protein A/G dynabeads cross-linked with anti-apolipoprotein antibodies, and after extensive washing, the resulting immunocaptured material was eluted from the beads, fractionated on 15% SDS-PAGE gels, and immunoblotted to detect α-syn (Fig. 3). As positive controls for detection of α-syn, 50 ng of recombinant α-syn (R-α-syn) and 10 μl of diluted plasma were also included on the gel. Plasma was also incubated with unconjugated beads as a control for non-specific binding of plasma proteins to the beads in the absence of capture antibody. Antibody-coupled beads were also incubated with PBS to control for elution of non-plasma components (e.g., antibody heavy and light chains) from the beads. The results show that α-syn is co-immunocaptured by apoE, apoA1, and apoJ antibodies, but not with anti-apoB antibody, as shown in Fig. 2.

**Fig. 4:**
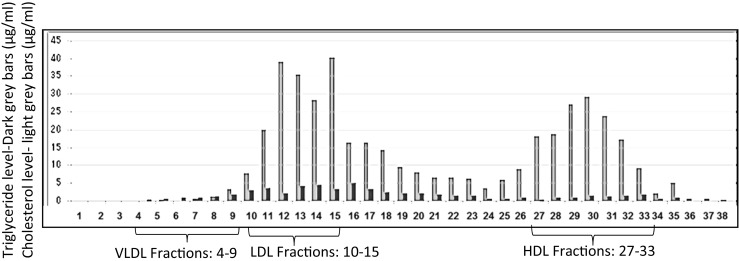
The chart shows which fractions are related to VLDL, LDL, and HDL, based on size exclusion chromatography and cholesterol analysis

To shed more light on possible associations between α-syn and lipoproteins, an alternative approach was taken by fractionating plasma into different lipoprotein components by gel filtration chromatography, followed by detection of α-syn in these fractions by ELISA and by western blotting. The size-fractionated samples of human plasma were prepared by utilizing two Superose-6 10/300GL gel filtration columns connected in tandem, to provide sufficient separation power for the lipoprotein fractions. The lipid (cholesterol and triglyceride) content of the 70 individual 0.5-ml fractions eluted from this system was determined, to help identify the lipoproteins present. Figure 4 shows separation of VLDL (fractions 4–9), LDL (fractions 10–15), and HDL (fractions 27–33) components from this column.

**Fig. 5:**
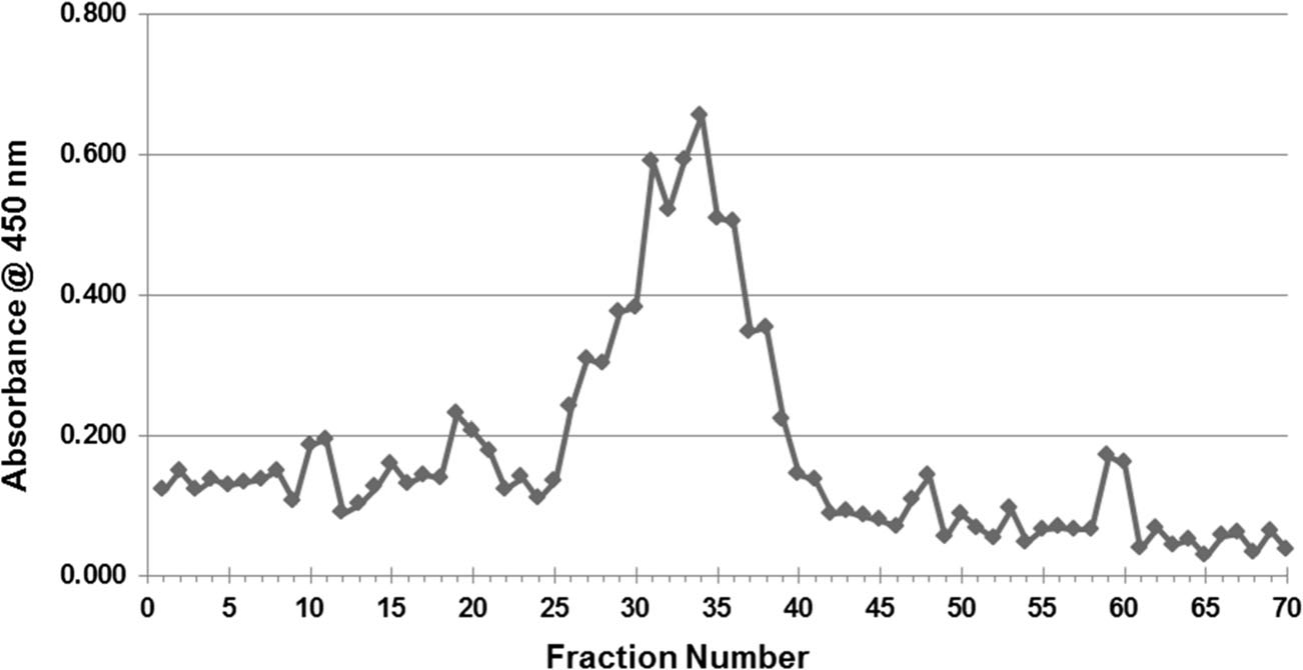
Measured ELISA absorbance values for α-syn from fractionated plasma samples. The same ELISA system as presented above was used to detect α-syn in the blood plasma fractions eluted from the tandem Superose-6 10/300GL column system. Most of the α-syn appears to be present in fractions 25–39, but there are also some smaller peaks present

**Fig. 6:**
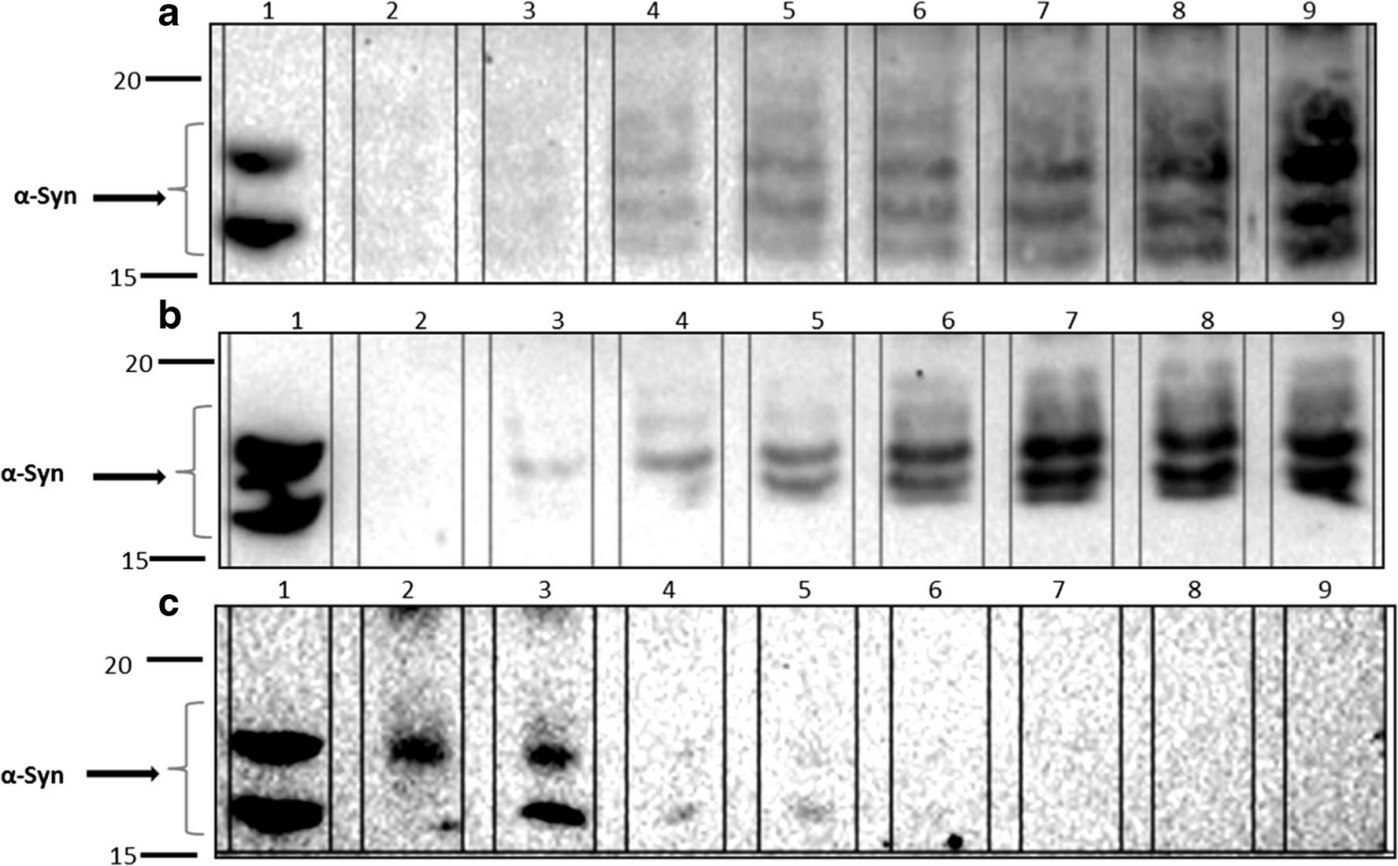
Fractions 17–40 were fractionated on a 12% SDS-PAGE gel and probed with anti-α-syn C211 antibody (1:1000). **a** Lane 1 = R-α-syn as a positive control. Lanes 2–9 are fractions 17–24. **b** Lane 1 = R-α-syn as a positive control. Lanes 2–9 are fractions 25–32. **c** Lane 1 = R-α-syn as a positive control. Lanes 2–9 are fractions 33–40

**Fig. 7:**
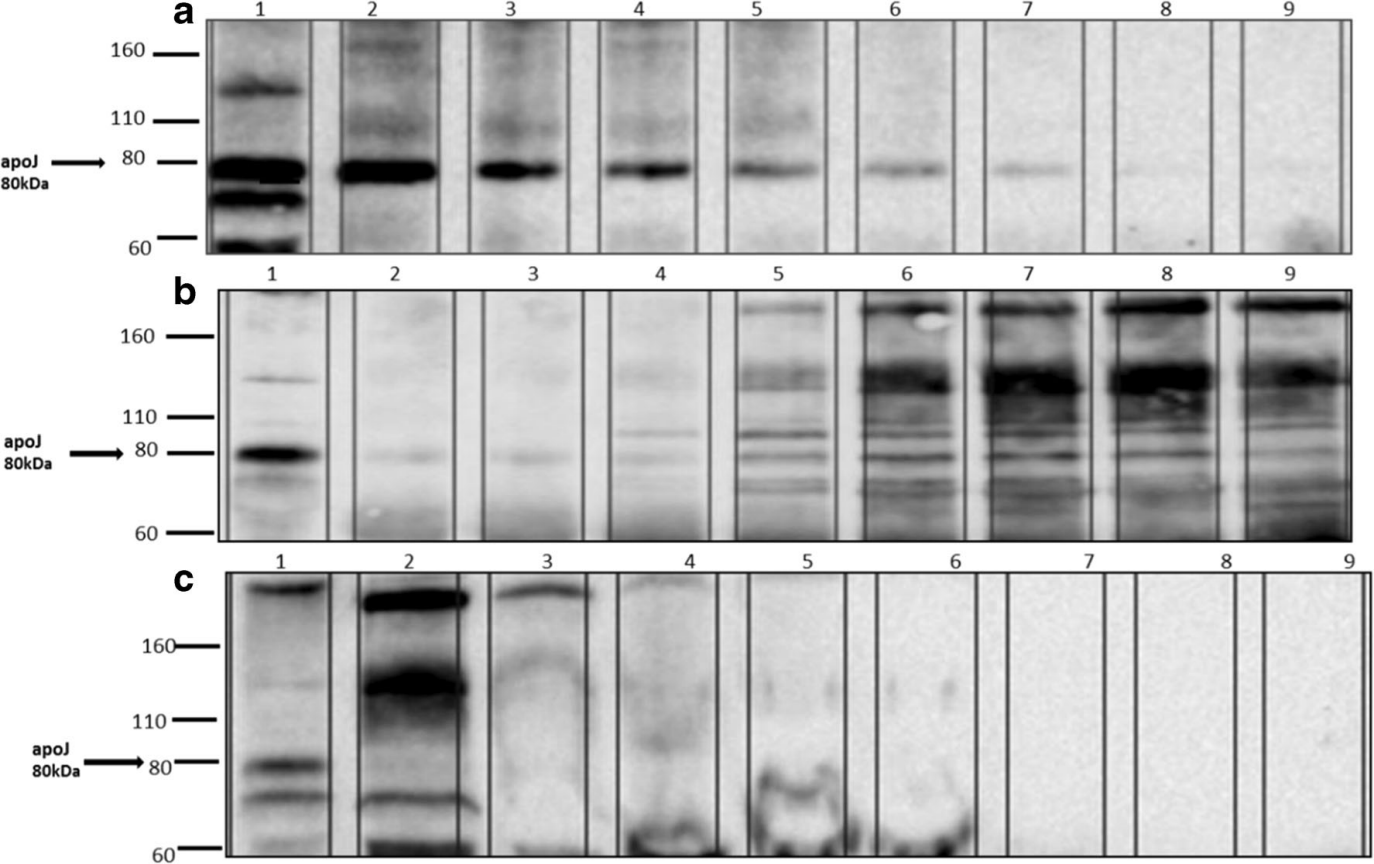
Fractions 17–40 were fractionated on a 12% SDS-PAGE gel and probed with anti-apoJ antibody (1:5000). **a** Lane 1 = whole plasma as a positive control. Lanes 2–9 are fractions 17–24. **b** Lane 1 = whole plasma as a positive control. Lanes 2–9 are fractions 25–32. **c** Lane 1 = whole plasma as a positive control. Lanes 2–9 are fractions 33–40

A sandwich ELISA for α-syn revealed that most of this protein was detected as a large and broad peak covering fractions 25–39 (Fig. 5), possibly with some other much smaller peaks on either side of this broad peak. When all 70 plasma fractions were subjected to SDS-PAGE/immunoblotting, employing anti-α-syn C211 antibody, α-syn was detected in fractions 19–24 relevant to the intermediate fraction between HDL and LDL (Lp(a)) and 27–34 relevant to HDL fractions and mainly in fractions 23–24 and 30–32 (Fig. 6). Fractions 9–40 from the plasma gel filtration experiment, which include those fractions that contain α-syn, were analyzed by SDS-PAGE/immunoblotting to determine which apolipoproteins are present, by employing anti-apoE, anti-apoA1, anti-apoJ, and anti-apoB antibodies (all diluted to 1:5000 in PBS-T). ApoE was present in all of these fractions. ApoA1 was also present in all of these fractions, although fractions 9–11 gave only faint bands, and fractions 21–24 and 31–34 had more intense bands. Figure 7 shows that apoJ was mainly present in fractions 17–21 that are related to (Lp(a)) and fractions 28–31 that are HDL-containing fractions.

## Discussion

α-Syn could interact with either the lipid components or the apolipoprotein components of lipoproteins. A potential interaction with lipids is strongly suggested by the fact that the N-terminal and C-terminal regions of α-syn can bind to fatty acids (Sharon et al. 2001) and α-syn can also interact with the cholesterol component of lipoproteins via its cholesterol-binding domain (Fantini et al. 2011). The potential role of α-syn in brain cholesterol homeostasis (Barceló-Coblijn et al. 2007), together with these lipid-binding properties of α-syn (Eichmann et al. 2016; Varkey et al. 2013), encouraged us to examine a possible association between α-syn and lipoprotein particles in blood plasma.

The immunoprecipitation experiment showed that α-syn appears to interact either directly or indirectly with apoA1, apoE, and apoJ. All of these apolipoproteins are found in the HDL sub-fraction of lipoproteins. ApoA1 is one of the exchangeable apolipoproteins that are actively involved in the regeneration of neuronal cells after injury, and CSF apoA1 levels have been shown to increase with the severity of macaque brain injury (Merched et al. 2000). ApoA1, as the main component of HDL, is also necessary for cholesterol transportation in the brain, and the plasma level of apoA1 is lower in PD patients than that in normal individuals, indicating a possible role for apoA1 in the pathogenesis of PD (Swanson et al. 2015; Qiang et al. 2013; Wang et al. 2010). We hypothesize that HDL, containing apoA1, may be involved in the transport of α-syn out of the brain. The gel filtration results also revealed that α-syn was detected in the fractions containing high levels of apoA1 and apoJ. These fractions are also related to HDL, based on size exclusion chromatography and cholesterol analysis.

Moreover, we found that α-syn was also present in the intermediate fractions between HDL and LDL. The intermediate fraction between HDL and LDL is referred to as lipoprotein (a) (Lp(a)) and is a risk factor for cardiovascular disease. Lp(a) has an important role in cholesterol metabolism, and its elevated levels lead to elevated cholesterol (Nordestgaard et al. 2010). Regulation of cholesterol metabolism in the CNS is important for proper function of brain cells, and any deficit in cholesterol can lead to many types of neurological disorders (Cermenati et al. 2013). Therefore, the presence of α-syn in the intermediate fractions between HDL and LDL could be due to an interaction between α-syn and the high amount of cholesterol contained in the Lp(a) fractions. This would be consistent with the presence of a cholesterol-binding domain at residues 67–78 of α-syn (Fantini and Barrantes 2013). We could also detect a large amount of ApoJ in the Lp(a) fractions, suggesting a possible role for apoJ in the trafficking of α-syn through the BBB. ApoJ is mainly produced by astrocytes and together with apoE is more abundant in the brain than any other apoprotein, and both interact with LRP (Vitali et al. 2014; Wang and Eckel 2014). Moreover, apoJ can also cross the BBB and is involved in reverse cholesterol transport and can efflux excess cholesterol from cells and deliver it back to the liver as a component of HDL (Gelissen et al. 1998). These data suggesting that α-syn can be detected in the Lp(a) plasma fraction are intriguing and should be investigated further.

Taking all of the results together, our data provide support for an association between α-syn and HDL, with a possible association with intermediate fractions between HDL and LDL, possibly Lp(a), and this could have important implications for transport of a-syn in the blood, and across the BBB.

α-syn, α-Synuclein; Apo, apolipoproteins; BBB, blood-brain barrier; CNS, central nervous system; CSF, cerebrospinal fluid; ELISA, enzyme-linked immunosorbent assay; LB, Lewy bodies; LRP1, low-density lipoprotein receptor-related protein-1; PD, Parkinson’s disease; SN, substantia nigra
